# QTLs Identification for Iron Chlorosis in a Segregating Peach–Almond Progeny Through Double-Digest Sequence-Based Genotyping (SBG)

**DOI:** 10.3389/fpls.2022.872208

**Published:** 2022-05-31

**Authors:** Verónica Guajardo, Pedro José Martínez-García, Simón Solís, Aitziber Calleja-Satrustegui, Christopher Saski, María Ángeles Moreno

**Affiliations:** ^1^Centro de Estudios Avanzados en Fruticultura (CEAF), Rengo, Chile; ^2^Department of Plant Breeding, Centro de Edafología y Biología Aplicada del Segura - Consejo Superior de Investigaciones Científicas (CEBAS-CSIC), Murcia, Spain; ^3^Department of Pomology, Estación Experimental de Aula Dei - Consejo Superior de Investigaciones Científicas (EEAD-CSIC), Zaragoza, Spain; ^4^Department of Science, Institute for Multidisciplinary Research in Applied Biology-IMAB, Universidad Pública de Navarra, Pamplona, Spain; ^5^Department of Plant and Environmental Sciences, Clemson University, Clemson, SC, United States

**Keywords:** molecular markers, rootstock, stone fruits, segregating population, reference genome, pseudomolecule, SPAD

## Abstract

Linkage maps are highly appreciated tools for cultivar and rootstock breeding programs because they are suitable for genetic and genomic studies. In this study, we report on using sequence-based genotyping (SBG) approach to simultaneously discover and genotype SNPs from two peach-based rootstocks (“Adafuel” and “Flordaguard”) and their progeny (*n* = 118): from a initial mapping population composed of 131 seedlings. The plant material was developed at the EEAD–CSIC *Prunus* rootstocks breeding program, aiming to obtain a segregating progeny for a range of characters of agronomical interest to rootstock breeding (iron-chlorosis and root-asphyxia tolerance, nematode resistance, vigor traits, and other effects on scion cultivars). Sequence reads obtained from double-digest SBG were aligned to the *P. persica* reference genome (Peach v2.0). While eight linkage groups were constructed for “Adafuel,” only four linkage groups were constructed for “Flordaguard,” given the low heterozygosity of this last genotype. High synteny and co-linearity were observed between obtained maps and Peach v2.0. On the other hand, this work aimed to elucidate the genetic basis of leaf chlorosis tolerance using the phenotypic segregation of the progeny to iron-chlorosis tolerance, along with the QTLs responsible for leaf chlorosis. The F1 mapping population, composed initially of 131 seedlings, was growing in four field trials established on calcareous soils at the experimental field of the EEAD-CSIC in Zaragoza, Spain. From the initial mapping population, 131 individuals were selected for their phenotypical characterization with SPAD measurements of plants grown in the field, exhibiting a great variability. Significant QTLs associated with tolerance to iron chlorosis were found in LG1, LG5, LG7, and LG8. The significant QTLs detected in LG5 and LG7 have not been associated with this abiotic stress before in *Prunus*. Several candidate genes such as Prupe.1G541100, predicted as glutamyl-tRNA reductase 1, Prupe.1G468200, encoding a 2-oxoglutarate (2OG), and Fe(II)-dependent oxygenase superfamily protein or Prupe.1G577000 (ppa011050.m), a NIFU-like protein 2 (NIFU2) were detected. The exact biological function of some of these genes should be verified for the future development of marker-assisted selection for peach iron chlorosis tolerance.

## Introduction

*Prunus* is one of the largest genera of the family *Rosaceae* and subfamily Prunoideae (Rehder, 1940). It is economically important as a source of edible and highly appreciated fruits (e.g., peaches, plums, cherries, and almonds) but also as a source of rootstocks for stone fruits production. Modern fruit trees are currently grafted onto a rootstock (Guajardo et al., 2020). The rootstock not only allows scion cultivars to be adapted to unfavorable soil conditions but also determines tree size, vigor, and fruit quality (Font i Forcada et al., 2020). Biotic and abiotic stress tolerance related to soil characteristics is mainly determined by rootstocks (Cummins and Aldwinckle, 1983; Layne, 1987). While new stone fruit scion cultivar development is addressed by many breeding programs around the world, few of them are dedicated to the selection of new *Prunus* rootstocks (Byrne et al., 2012).

One of the main objectives of these breeding programs for rootstocks is to generate complex hybrids with the maximum number of desirable traits. The new rootstocks should overcome soil and disease problems (Reighard, 2002), showing high-performance and excellent adaption to different regions such as the Mediterranean area. One worldwide abiotic stress presented in these regions is iron chlorosis (Marschner, 1995). This abiotic stress is mainly due to the low iron bioavailability in calcareous soils and the difficulty of iron (Fe) acquisition by the roots (Hell and Stephan, 2003). The selection of rootstocks tolerant to iron chlorosis requires expensive and long-term experiments (Zarrouk et al., 2005; Jiménez et al., 2008). According to Gonzalo et al. (2012), the SPAD (Soil Plant Analysis Development) value seems to be the most reliable measure, and an unbiased and non-destructive method to evaluate the tolerance to iron chlorosis of different genotypes and breeding populations. The selection process can be also accelerated using molecular markers linked to the trait, allowing the early selection of likely seedlings (Etienne et al., 2002; Dirlewanger et al., 2006). Combining SPAD value and low number of molecular markers, Gonzalo et al. (2012) identified different quantitative trait loci (QTL) in linkage groups (LG) 4 and 6 in the almond–peach hybrid “Felinem” (*Prunus dulcis* × *Prunus persica*), formerly named “GxN 22” (Felipe, 2009). Although this work allowed localizing the candidate gene PFIT in LG 4, the genetic control of iron chlorosis in *Prunus* is still unknown. In fact, other important candidates such as PIRT1, PAHA2, and PNramp were not located by the authors (Gonzalo et al., 2012).

At present, new advances in genetic and genomic tools can help to face challenges in both cultivar and rootstocks breeding programs by improving the efficiency and reducing the time necessary to obtain new genotypes. In this sense, high-resolution linkage maps are valuable tools in genetics and breeding because they are the basis for QTLs and the identification of genes associated with traits of interest. For *Prunus* rootstocks, traditionally linkage maps have been constructed using amplified fragment length polymorphism (AFLPs) markers (Lu et al., 1998), AFLP and single sequence repeat (SSRs) markers (Blenda et al., 2007), and SSRs only (Gonzalo et al., 2012). In the last decade, the use of molecular markers such as single nucleotide polymorphisms (SNPs) has allowed the construction of high-density linkage maps for *Prunus* cultivars, but their use in linkage maps construction for rootstocks is scarce. For example, mapping populations have been genotyped using SNP chips (Peace et al., 2012; Verde et al., 2012) for the construction of linkage maps in peach (Eduardo et al., 2013; Martínez-García et al., 2013; Pirona et al., 2013; Yang et al., 2013; Frett et al., 2014; Romeu et al., 2014; da Silva et al., 2015, 2018; Donoso et al., 2015; Núñez-Lillo et al., 2015; Fresnedo-Ramírez et al., 2016; Lambert et al., 2016; Zeballos et al., 2016; Serra et al., 2017), sweet cherry (Klagges et al., 2013; Campoy et al., 2015; Calle et al., 2018), and sour cherry (Cai et al., 2018).

More recently, *Prunus* linkage maps have been constructed using SNPs identified through sequence-based genotyping (SBG, Elshire et al., 2011), a next-generation sequencing methodology that provides a reduced representation of the genome by using single and/or double-digestion of DNA with restriction enzymes to reduce genome complexity and genotype multiple DNA samples (Elshire et al., 2011; Poland et al., 2012). Single digestion using *Ape*KI restriction enzyme has been used for *Prunus* linkage maps construction in peach (Bielenberg et al., 2015; Núñez-Lillo et al., 2019), sweet cherry (Guajardo et al., 2015), Japanese plum (Salazar et al., 2017; Carrasco et al., 2018), almond (Goonetilleke et al., 2018), *P. mume* (Kitamura et al., 2018), and apricot (Pina et al., 2021). Although good genome coverage has been reported using this methodology, it has been described that the double-digest protocol greatly simplifies quantification of the library prior to sequencing and could generate a suitable and uniform complexity reduction of the genome (Poland et al., 2012; Guajardo et al., 2020).

In this study, we used the double-digest SBG for the identification of SNPs and to construct linkage maps for a mapping population derived from the cross “Adafuel” × “Flordaguard,” two *Prunus* rootstocks. Filtered sequence reads were aligned to the version 2.0 of the *Prunus* reference genome (Verde et al., 2017). The physical position of each SNP was determined, and the distribution of SNPs along eight peach pseudomolecules was performed to analyze the location of each SNP, identifying SNP-carrying genes and predicting the SNP effect of each SNP. Linkage maps constructed for both peach-based rootstocks were used for the identification of QTLs for iron chlorosis evaluated during two years in the mapping population. The results obtained here will provide a list of genes relevant for *Prunus* rootstocks breeding.

## Materials and Methods

### Plant Material

The mapping population was comprised of 118 (of the total 131) seedlings obtained from the cross between the peach–almond rootstock “Adafuel” (*P. dulcis* × *P. persica*; 2n = 2x = 16; Cambra, 1990) and the peach-based rootstock “Flordaguard” (*P. persica*; 2n = 2x = 16; Sherman et al., 1991). “Adafuel” is an almond × peach hybrid rootstock selected from an open-pollinated population of *P. dulcis* cv. Marcona for adaptation to calcareous and unfavorable soils for peach growing (Moreno et al., 1994). It is tolerant to iron chlorosis and susceptible to root asphyxia (Mestre et al., 2015). In turn, “Flordaguard” is a red leaf peach rootstock, a sixth-generation descendant from the cross Chico 11 × *P. davidiana* (Carr.) Franch, C-26712. Chico 11 was a seedling of the ornamental *P. persica* cv. Shau Thai, PI 65821 (Sharpe et al., 1969). After selection for nematode resistance, “Flordaguard” was finally released (Sherman et al., 1991). “Flordaguard” is very sensitive to iron-chlorosis and could be more tolerant to root asphyxia than “Adafuel.” The segregating population was established at the experimental trail (block B1) of the EEAD-CSIC in Zaragoza (North-Eastern of Spain; lat. 41° 43′ 42.7″ N, long. 0° 48′ 44.1″ W). To ensure enough replications for iron chlorosis analyses, at least 10 clonally propagated plants of each genotype (considered as an individual plot inside of each block) were planted in three additional experimental trials (B2, B3, and B4) of the EEAD-CSIC, established on calcareous soil having 29–31% total calcium carbonate, 8.1–9.9% active lime, pH 8.5, and a silt-loam texture.

### Population Phenotyping

The iron chlorosis level was assessed with a SPAD-502 Chlorophyll Meter (Minolta Co., Osaka, Japan) in ungrafted clonally propagated plants of 131 genotypes grown in the field. The chlorophyll (Chl) concentration per unit leaf area was estimated using 10 leaves per tree, selected from the middle of bearing shoots located all around the crown, to obtain an average leaf value representative of the leaves belonging to the outer part of the tree canopy. SPAD measurements were carried out ~120 days after full bloom in the four experimental trials (blocks) for two years. For each genotype, at least three ungrafted trees were assessed in each experimental trial.

A mixed model was developed to represent our experimental design and to obtain the adjusted means (lsmeans) using the procedure PROC MIXED within SAS (SAS Institute Inc, 2017). The calculated lsmean for each individual tree was used in the QTL analysis. The mixed model was:


(1) $$\begin{array}{r} Y\text{\_}ikm=\mu +\;\alpha \text{\_}i+\gamma \text{\_}m+\left(\alpha \gamma \right)\text{\_}im+\beta \text{\_}ik+\;\varepsilon \text{\_}imk \end{array}$$

where Y_ikm is the mean value per plot of clone i in block k and year m, μ is the intercept, α_i and γ_m are the fixed effects of the m-th clone and i-th year, (αγ)_im the random effect of the i-th clone error at the m-th year, β_ik is the random effect of the k-th block within the i-th year, and ε_imk is the effect of the error of plot mean y_ikm. For all random effects including the error, year-specific variances and an additional covariance were fitted, if these extensions increase the model *via* AIC (Akaike, 1974).

### DNA Extraction

Genomic DNA was extracted from young leaves collected from the parents and seedlings and stored at−80°C until use. The homogenization of leaf samples was reached after grinding around 100 mg of tissue with the Mixer Mill MM 400 (Retsch, Germany), and the tubes were immersed in liquid nitrogen to freeze the tissue. The NucleoSpin Plant II kit (Macherey-Nagel, Germany) was used for DNA extraction according to the manufacturer's instructions using the Lysis Buffer PL1, which is based on the CTAB method. DNA was eluted in 50 μL buffer PE and it was quantified using spectrophotometry (Tecan Trading AG, Switzerland). DNA quality was determined using 1% agarose electrophoresis.

### Sequence-Based Genotyping (SBG)

Sequence-based genotyping was carried out at Clemson University Genomics Computational Laboratory (CUGCL; Clemson, SC, USA). A reduced representation SBG library was prepared using restriction enzymes, *Pst*I (methylation sensitive) and *Msp*I (partial sensitivity to methylation), as described by Poland et al. (2012). A total of 200 ng of intact genomic DNA was digested and ligated to custom-designed adapter sequences. A total of 120 SBG libraries were sequenced on an Illumina HiSeq2500 using a 2 ×125 bp paired-end read module across 2 high-output lanes. Raw sequence data were demultiplexed and preprocessed for errors using the Stacks demultiplex tool (Catchen et al., 2013). Sample specific sequences were aligned to the eight pseudomolecules representing the eight chromosomes of the peach genome assembly (Peach v2.0; Verde et al., 2017) with the GMAP/GSNAP release 816.16 (Wu et al., 2016). The resulting variant call file (.vcf) was filtered for SNPs with a minimum depth (DP) of six, and present in at least 80% of the samples. Mean coverage of each SBG SNP was determined by creating a.BED file from the final SNP set and generating a bed graph with the genomecov function of bedtools v. 2.28.0 (Quinlan and Hall, 2010), and intersecting the bedgraph with the SNP.bed file with the intersect function in bedtools. The mean coverage of each sample was determined with in-house scripts. SNPs were extracted using the pipeline implemented in TASSEL 5.2.5 software (Bradbury et al., 2007), and accessions were called using minor allele frequency (MAF) > 0.05. SNPs were labeled according to the pseudomolecules in the peach genome (Pp01 to Pp08), followed by the physical position in base pairs (bp).

### Linkage Maps Construction

Markers scoring followed the coding scheme for cross-pollinated population type proposed in JoinMap 4 (Kyazma B.V., Netherlands; Van Ooijen, 2006) and the evaluation version of JoinMap 5 (https://www.kyazma.nl/index.php/JoinMap/Evaluate). Linkage maps were constructed following the protocol described for sweet cherry by Guajardo et al. (2015). Markers with identical genotypic scores (Similarity of loci = 1), which are automatically removed by JoinMap because they should be mapped to the same position on the linkage group, were added back to the resulting linkage maps. Markers showing segregation distortion were also integrated into the maps and they were plotted using MapChart 2.3 (Voorrips, 2002). For a comparison between physical and genetic distance of mapped SNPs, the genetic positions of each SNP (in centimorgans, cM) were plotted against their physical position on the Peach v2.0 (in Megabase pairs, Mbp). Results were compared with those obtained by Verde et al. (2017) for the peach selection IF7310828 × Ferganensis BC1 (PxF) population using the IPSC 9 K SNP array (Verde et al., 2012) with the physical position of each SNP updated with the Peach v2.0 as a reference genome (Verde et al., 2017).

### QTL Analysis of Iron Chlorosis Tolerance

Quantitative trait loci analysis of ion chlorosis was performed with MapQTL® 6.0 (Van Ooijen, 2009) using SPAD data from each individual for the two seasons evaluated. According to the no assumption of normality distribution of the data, the rank-sum test of Kruskal–Wallis (K-W; test non-parametric equivalent of the one-way analysis of the variance) was performed for the trait against each SNP, separately, to establish the strength of linkage at individual SNP loci. The K-W test ranks all individuals according to the quantitative trait, while it classifies them according to their marker genotype. A stringent significance level (*P*-value) similar or higher than 0.005 was used to identify markers significantly associated with both traits by the K-W test (Van Ooijen, 2009).

### Structural and Functional Characterization of SNPs

The physical position of each SNP was used to identify common markers in this study and the IRSC 9K peach SNP array v1 (Verde et al., 2012), which was updated with the Peach v2.0 as a reference genome (www.rosaceae.org; Verde et al., 2017). The prediction of SNP effect, based on annotated genes or their genomic locations, was performed using the SnpEff v4.3 program (Cingolani et al., 2012) with Peach v2.0 as reference. Whenever multiple transcripts for a gene exist, the effect on each transcript was analyzed, which was the same criteria applied by Guajardo et al. (2020) for SNPs identified from a vast group of *Prunus* rootstocks, where “Adafuel” and “Flordaguard” were also included. The SNP predicted effects were categorized by impact, as high (disruptive impact on the protein), moderate (non-synonymous substitution), low (synonymous substitution), or modifier (with impact on noncoding regions). To examine the putative function of the genes containing high impact SNPs, a eukaryotic orthologous group (KOG) analysis was carried out using tools from Join Genome Institute (JGI, https://jgi.doe.gov). Information about GO, PFAM and Phanter for each gene of this group was obtained from Phytozome (https://jgi.doe.gov).

## Results

### Phenotyping

**Figure 1:**
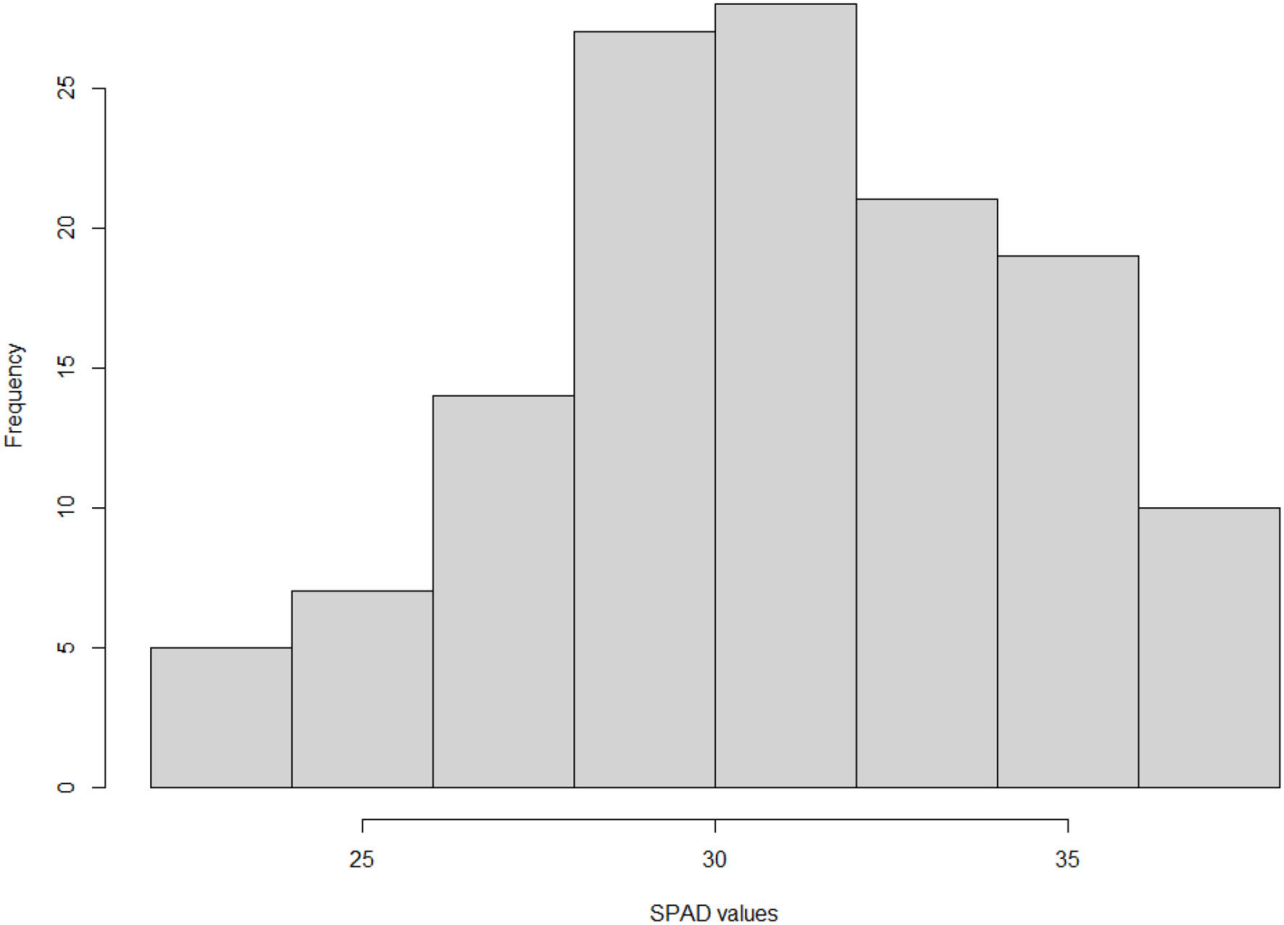
Frequency distribution of Lsmeans for SPAD values of 131 peach–almond individual hybrids from the cross of “Adafuel” × “Flordaguard” using the hist() function in R programming language (R Core Team, 2019).

Different measurements were implemented to guarantee an accurate data set for the genetic analysis of 131 genotypes, clonally propagated and growing in three experimental fields. Leaf chlorophyll content, used as a good indicator of leaf chlorosis in trees, was estimated by using a SPAD meter. According to the Lsmeans obtained from our model, the values showed a great segregation response and significant differences between genotypes (*p*-value <0.001). Lsmeans ranged significantly among genotypes and years from 22.013 to 37.40 (Figure 1). No significant difference was observed between years (*p*-value 0.2753).

### High-Throughput Genotyping of the Mapping Population

Double-digest SBG produced between 4,293,116 and 18,572,896 read pairs, with an average of 11,321,053 reads per individual. This deep sequencing led to a mean depth per SNP of 1,244 across the entire dataset (Supplementary Table 1). The number of unique tags varied between 1,548,906 and 6,620,490, with an average of 3,990,190 tags per individual.

A total of 18,912 high quality SNPs (MAF > 0.05; missing data <5%), evenly distributed along the eight peach pseudomolecules, were used for mapping (Table 1). The number of identified SNPs ranged from 1,595 for Pp07 to 4,042 for Pp01. A total of 224.3 Mbp (99.4%) of the peach genome was covered with a marker density of ~1 SNP per 11.9 Kbp. Gaps were observed in all pseudomolecules, with the largest gap per pseudomolecule ranging from 506 Kbp (Pp06) to 1.2 Mbp (Pp01 and Pp04).

**Table 1:** Physical position of SNPs identified by sequence-based genotyping (SBG).

**Pseudomol**.	**Size (bp)**	**No. SNPs**	**Physical position of the first SNP (bp)**	**Physical position of the last SNP (bp)**	**Pseudomol. coverage (bp)**	**Pseudomol. coverage (%)^a^**	**Pseudomol. coverage (bp)/ No. SNPs**	**Maximum gap (bp)**	**Last SNP before the maximum gap**	**First SNP after the maximum gap**
Pp01	47,851,208	4,042	60,532	47,767,955	47,707,423	99.7	11,802.9	1,175,195	21,127,025	22,302,220
Pp02	30,405,870	2,337	202,304	30,219,724	30,017,420	98.7	12,844.4	963,246	9,920,338	10,883,584
Pp03	27,368,013	2,296	79,029	27,263,085	27,184,056	99.3	11,839.7	818,148	11,905,349	12,723,497
Pp04	25,843,236	2,194	100,499	25,834,933	25,734,434	99.6	11,729.5	1,183,973	20,244,461	21,428,434
Pp05	18,496,696	1,827	140,802	18,416,175	18,275,373	98.8	10,002.9	901,131	6,809,924	7,711,055
Pp06	30,767,194	2,658	144,309	30,738,182	30,593,873	99.4	11,510.1	506,234	14,972,607	15,478,841
Pp07	22,388,614	1,595	1,873	22,314,985	22,313,112	99.7	13,989.4	648,099	4,394,737	5,042,836
Pp08	22,573,980	1,963	43,718	22,543,158	22,499,440	99.7	11,461.8	1,106,932	7,280,561	8,387,493
**Total**	225,694,811	18,912			224,325,131					
**Average**						99.4	11,897.6			

a *Pseudomolecule coverage based on lenght on peach pseudomolecules (Verde et al., 2017)*.

### Linkage Mapping

**Figure 2:**
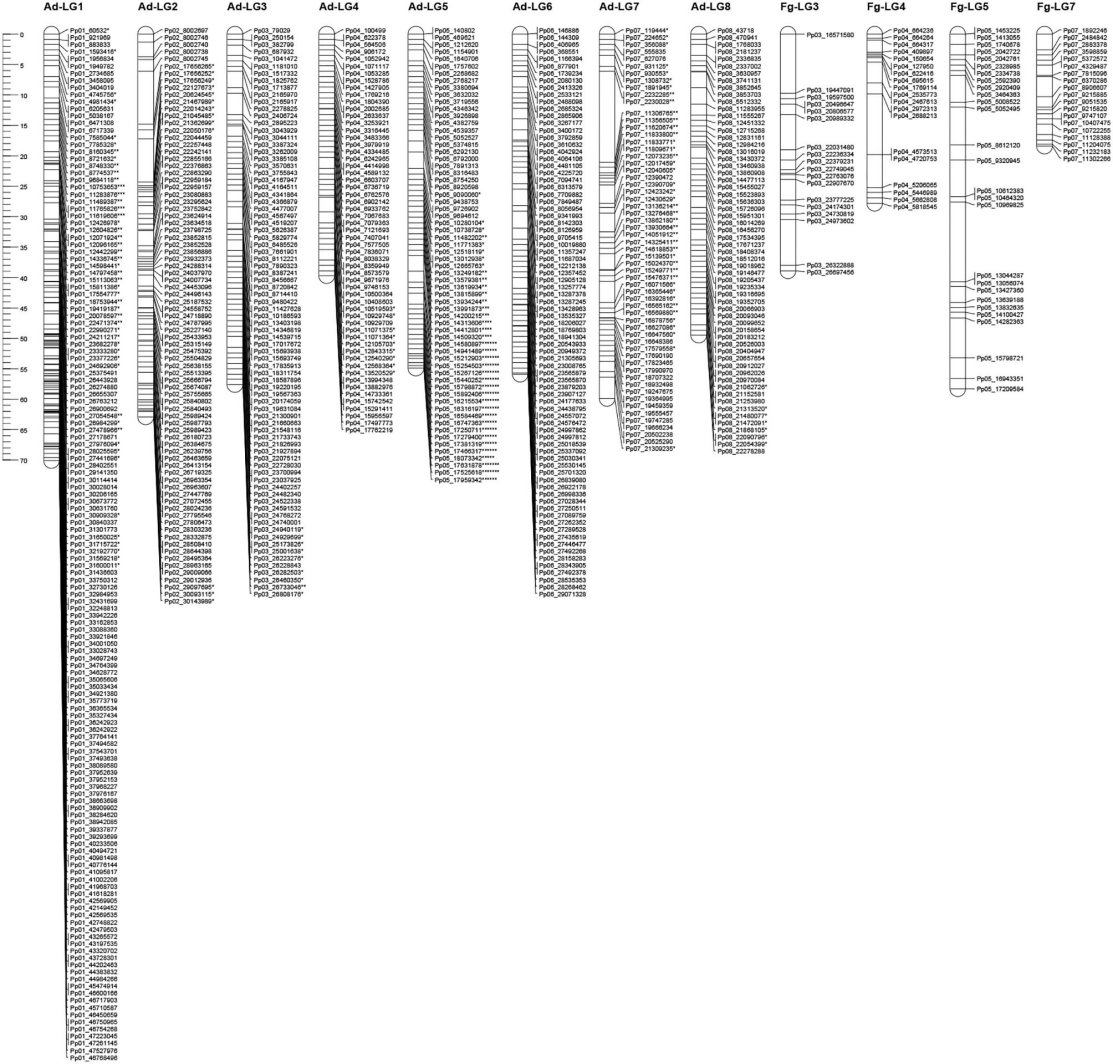
Linkage maps for “Adafuel” and “Flordaguard.” Asterisks indicate distortion level of skewed markers. *p < 0.1; **p < 0.05; ***p < 0.01; ****p < 0.005; *****p < 0.001; ******p < 0.0005; *******p < 0.0001. Redundant markers have been removed and only unique positions are represented. The scale in centiMorgan (cM) is given at the left side of the figure.

Out of the total 18,912 SNPs, 17,539 (92.7%) exhibited the maternal segregation type < lmxll>, 868 (4.6%) showed the paternal segregation type < nnxnp> and 505 (2.7%) were heterozygous for both parents, with segregation type < hkxhk>. The last group of SNPs was not used for linkage maps construction. The linkage map obtained for the “Adafuel” genotype was composed of eight linkage groups (Ad-LG 1–8; Figure 2 and Table 2) and was comprised of 626 uniquely mapped SNPs covering a total of 450.6 cM. Ad-LG1 was the largest linkage group with 70.1 cM and Ad-LG4 covered the shortest distance, 39.7 cM. The average marker density was 0.8 cM per marker. The maximum gap size ranged from 2.4 cM in Ad-LG4 to 14.9 cM in Ad-LG7. In contrast, the “Flordaguard” linkage map was composed just only with four linkage groups (Fg-LG3, Fg-LG4, Fg-LG5, and Fg-LG7, Figure 2). This map comprised of 79 uniquely mapped SNPs, covering a total of 142.7 cM (Table 2), with a minimum and maximum linkage group length of 18.4 cM for Fg-LG7 and 58.3 cM for Fg-LG5 and an average marker density of 1.8 cM per marker. The maximum gap size ranged from 3.3 cM for Fg-LG7 to 13.1 cM for Fg-LG5. When markers with identical genotypic scores were added back to the resulting linkage maps, a significant clustering of SNPs co-segregating at the same map positions in some linkage groups were observed (Table 2 and Supplementary Table 2). For example, while the number of uniquely mapped SNPs on Ad-LG1 was 145, the number of co-segregating SNPs was 3,865 on Ad-LG1, resulting in a total of 7,802 SNPs mapped on “Adafuel” linkage map and 225 SNPs mapped on “Flordaguard” linkage map. Furthermore, and considering all the linkage groups of “Adafuel” and “Flordaguard,” a high number of skewed markers (*p* < 0.01) was mapped in the bottom part of Ad-LG5 (Figure 2).

**Table 2:** Description of “Adafuel” and “Flordaguard” linkage maps.

	**“Adafuel”**					**“Flordaguard”**				
**LG**	**Mapped SNPs**	**Uniquely mapped**	**Lenght (cM)**	**Av. marker distance (cM)**	**Max. gap (cM)**	**Mapped SNPs**	**Uniquely mapped**	**Lenght (cM)**	**Av. marker distance (cM)**	**Max. gap (cM)**
1	3,865	145	70.1	0.5	2.7	Un	un			
2	1,357	81	62.9	0.8	8.8	Un	un			
3	2,127	80	57.6	0.7	3.5	34	18	38.9	2.2	9.7
4	62	57	39.7	0.7	2.4	53	15	27.1	1.8	10.1
5	181	64	55	0.9	2.7	46	27	58.3	2.2	13.1
6	87	80	55.9	0.7	2.8	Un	un			
7	63	59	59.9	1.0	14.9	92	19	18.4	1.0	3.3
8	60	60	49.5	0.8	2.7	Un	un			
**Total**	**7,802**	**626**	**450.6**	**0.8**		**225**	**79**	**142.7**	**1.8**	

*un, undetermined*.

**Figure 3:**
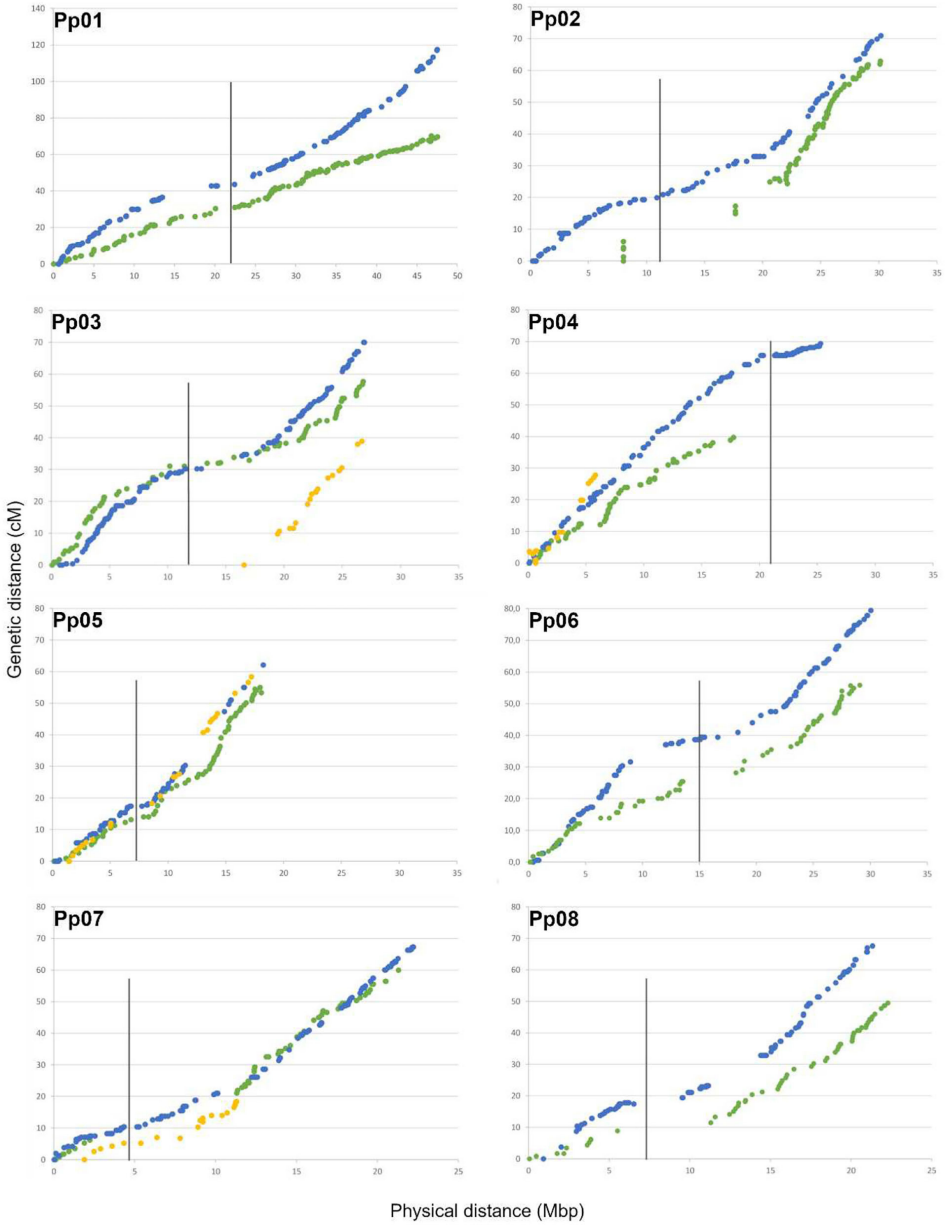
Relationship between genetic and physical distances for each linkage group and pseudomolecule. Physical distance along each pseudomolecule is shown on the horizontal axis (Mbp), and the genetic distance is shown on the vertical axis (cM). The location of markers from “Adafuel” (green), “Flordaguard” (yellow), and P × F (blue) linkage maps are shown. Redundant markers have been removed and only unique positions are represented. The vertical bars indicate the putative position of the centromeric regions using information from the Peach v2.0 (Verde et al., 2017).

The important gaps observed for some linkage groups were related to considerable physical distance between flanking SNPs (Table 2 and Figure 3). For “Adafuel,” overlapping gaps between genetic and physical maps were observed in Ad-LG2 (8.8 cM with 9.7 Mbp), Ad-LG7 (14.9 cM with 9.1 Mbp), and Ad-LG8 (2.7 cM with 5.8 Mbp), and for “Flordaguard,” in Fg-LG4 (10.1 cM with 1.9 Mbp), Fg-LG5 (13.1 cM with 2.1 Mbp), and Fg-LG7 (3.3 cM with 2.5 Mbp). The distribution of SNPs is similar to those observed for the P × F map (Figure 3), although the genetic distance covered by each “Adafuel” and “Flordaguard” linkage group was shorter when it was compared with P × F linkage groups.

**Figure 4:**
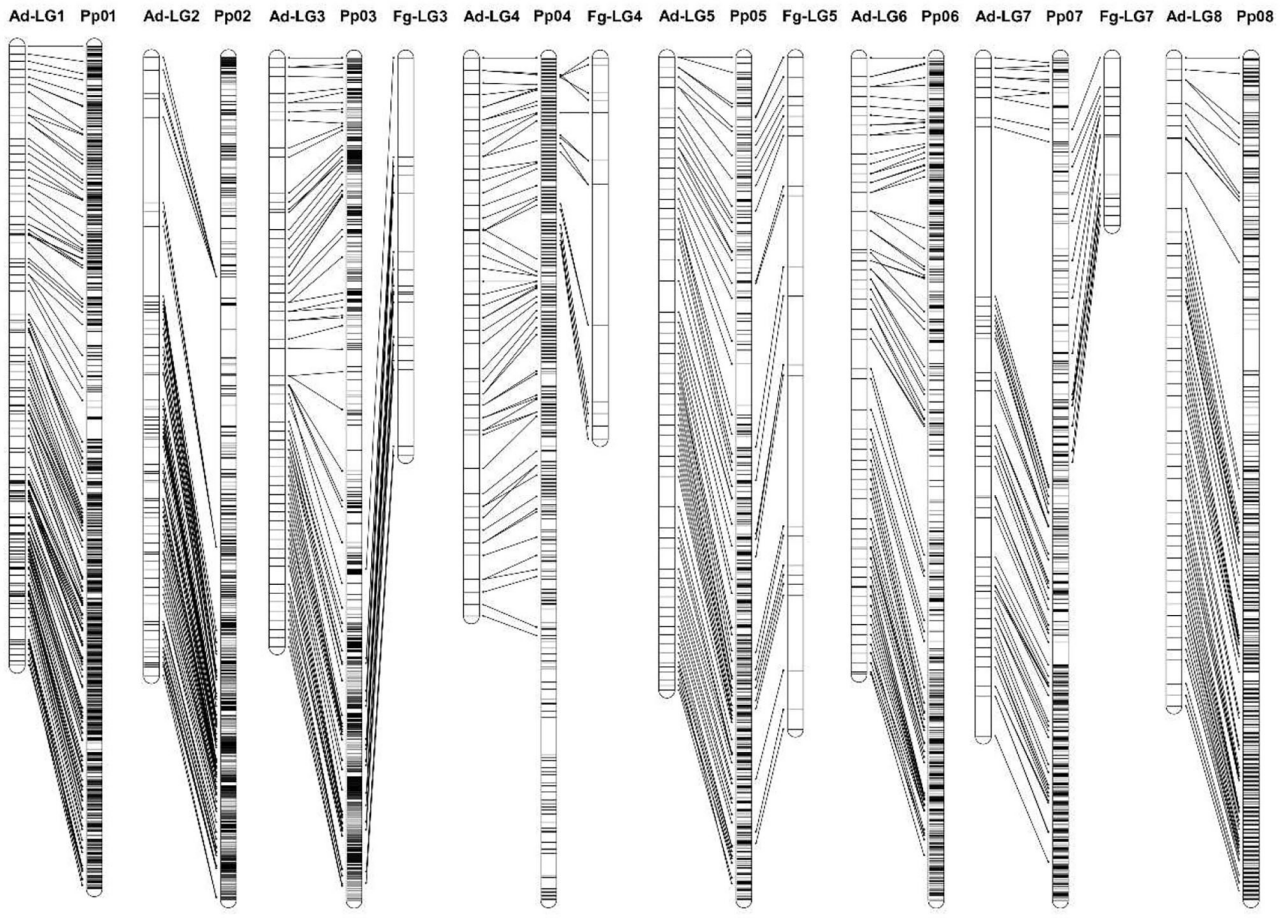
Comparison of the parental linkage maps and physical map. The physical map was constructed using all SNPs identified by GBS for the mapping population used in this study (*n* = 19,912) and ordered according to their physical position in the Peach v2.0 (Verde et al., 2017). Redundant markers have been removed and only unique positions are represented. Distances have been proportionally modified to show the results.

The linkage position of all SNPs mapped in “Adafuel” and “Flordaguard” linkage maps were in agreement with their physical position on the pseudomolecules of the Peach v2.0 (Figure 4). As it was previously indicated, it was possible to construct only four linkage groups for “Flordaguard,” and also partial coverage of pseudomolecules was observed for Fg-LG3, Fg-LG4, and Fg-LG7, with mapped SNPs only from the bottom region for Fg-LG3 and from the top region for Fg-LG4 and Fg-LG7.

### QTL Detection for Ion Chlorosis Tolerance

**Figure 5:**
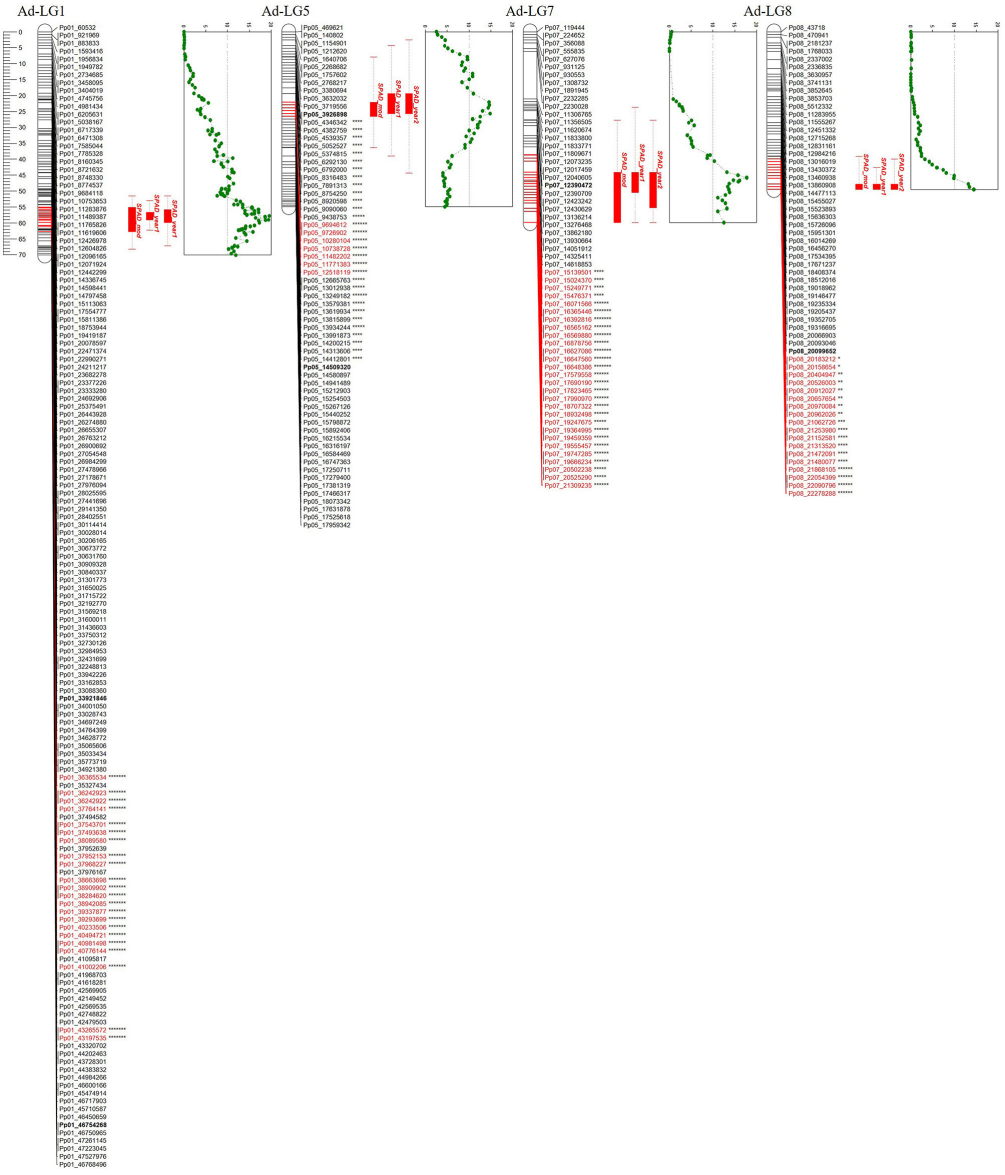
QTLs obtained for each year of the study, and the lsmeans obtained with the fitted model for iron chlorosis in “Adafuel” (Ad). At the right side of each linkage group, dotted green line shows QTL LOD value variation along the map and the likelihood peak of the QTL. Significant markers by Kruskal-Wallis test statistic in Ad-LG1, Ad-LG5, Ad-LG7, and Ad-LG8 are highlighted in red. In Ad-LG1, Ad-LG5, and Ad-LG7 only high levels of significance are shown. Asterisks indicate distortion level of skewed markers. *p < 0.1; **p < 0.05; ***p < 0.01; ****p < 0.005; *****p < 0.001; ******p < 0.0005; *******p < 0.0001.

To identify QTLs involved in iron chlorosis, analysis for each year and genotype was performed. The QTLs were placed on the genetic map of “Adafuel,” comprised of 626 uniquely mapped positions (SNPs), covering a total of 450.6 cM. The results obtained from the non-parametric Kruskal–Wallis for iron chlorosis revealed several QTLs in Ad-LG1, Ad-LG5, Ad-LG7, and Ad-LG8. The number of SNPs identified and the length of each QTL in each linkage group was different. In total, 1,263 SNP markers, located in 95 unique map positions, were strongly associated (from *p* = 0.05 to *p* = 0.0001) with this trait (Figure 5). In Ad-LG1, 1,192 SNPs were associated with SPAD, located mainly at the bottom of this linkage group and spanning a map distance of around 17 cM (between 51 and 68 cM). In Ad-LG1, the higher number of SNPs were mapped in three map positions at 55.27 cM, 58.11 cM, and 61.71 cM, with 205, 108, and 196 SNPs, respectively. In Ad-LG5, a total of 33 SNPs were significant, with a high LOD peak between 22 and 26 cM. In Ad-LG7, 29 SNPs were highly significant for SPAD, with a high LOD between 45 cM and 47 cM. At the bottom of Ad-LG8, a total of nine SNPs markers were highly associated with SPAD.

### SNP Effect Prediction

When mapped SNPs for the “Adafuel” × “Flordaguard” seedlings were analyzed using the physical position of each SNP along the eight pseudomolecules of the peach genome, a total of 23,626 functional effects for SNP variants were predicted for 8,027 SNPs (7,802 SNPs for “Adafuel” and 225 SNPs for “Flordaguard;” Supplementary Table 3). Also, a group of 5,586 different genes (or 20.8% of genes identified in the peach genome sequence) could be affected by this group of SNPs. The predicted effects were of modifier type (78.52%), low impact (13.72%), moderate impact (7.73%), and high impact (0.03%). For SNPs with a modifier effect, downstream gene variant was the most represented category (38.14%), while synonymous variant (89.13%) and missense variant (99.15%) was the most represented category for low and moderate effect, respectively. Fifteen genes were categorized as highly impacted (Supplementary Table 4).

For SNP effect analysis of those SNPs strongly associated with iron chlorosis according to the QTL identified in Ad-LG1, Ad-LG5, Ad-LG7, and Ad-LG8, both uniquely and co-segregating SNPs were considered. The predicted effects were of modifier type (79.20%), low impact (12.12%), moderate impact (8.62%), and high impact (0.05%). In Ad-LG1, a total of 3,522 effects were detected. The number of effects was lower in the rest of pseudomolecules, with 103 effects in Ad-LG5, 93 in Ad-LG7, and 28 effects in Ad-LG8. These effects affected a total of 970 unique peach (Prupe) genes, as commented before, the majority of them located in Pp01. Several annotations for homologs of these peach genes have been provided, and in total, this list of genes matches with 754 homologs *Arabidopsis* genes (Supplementary Table 5). A total of 621 unique descriptions were obtained for these genes. Only two genes on Ad-LG1 (Prupe.1G566900 and Prupe.1G570100) were categorized as highly impacted because they present a stop codon. In addition, complete information on candidate genes located in the QTL regions is provided (Supplementary Table 6).

## Discussion

In this work, a successful strategy covering the discovery of single nucleotide polymorphism (SNP) through double-digest sequence-based genotyping (SBG), genetic linkage maps construction, and QTL identification has been performed in a segregating peach–almond progeny to improve the knowledge about the genetic control of the tolerance to iron chlorosis in *Prunus*. The physical position and distribution of each SNP along the eight peach pseudomolecules were determined to identify SNP-carrying genes and to predict the effect of each SNP. To our knowledge, this is the first report on linkage map construction and QTL analysis using SNPs obtained from SBG for *Prunus* rootstocks.

Our results showed excellent performance for double digest-SBG and deep coverage paired-end sequencing for reduced representation of *Prunus* rootstocks. A high mean depth (1,244) per SNP and high coverage of peach pseudomolecules were reached, which was higher than a coverage of 12.1x obtained by Núñez-Lillo et al. (2019) for map construction in peach using single-digestion SBG. Our results confirm that paired-end sequence reads are more accurately mapped onto the reference genome, showing a significantly lower amount of missing data and a greater number of quality SNPs, as was suggested by Shirasawa et al. (2016) and Guajardo et al. (2020). In total, the SNP density was found to be ~1 SNP per 11.9 Kbp, which can be enough for mapping and QTL identification in an F1 population.

The length of “Adafuel” linkage map, composed by eight linkage groups, is comparable with other maps reported for peach cultivars (Martínez-García et al., 2013; Yang et al., 2013; Núñez-Lillo et al., 2015) and for almond x peach maps (Donoso et al., 2015). A high number of SNPs shared map positions in Ad-LG1, Ad-LG2, and Ad-LG3 (Table 2), probably due to the absence of recombination between some SNPs. On the other hand, and given the low number of SNPs showing paternal segregation (868 SNPs, or 4.6% of the useful SNPs for mapping), it was possible to construct only four linkage groups for “Flordaguard.” It might be due to the origin of this rootstock, which originated from six generations of peach crosses and open-pollinated seedlings (Sherman et al., 1991), generating low levels of heterozygosity in certain regions, or gaps, of the “Flordaguard” genome.

In general, for both “Adafuel” and “Flordaguard” linkage maps, the genetic positions of mapped SNPs are in agreement with the physical position of SNP along the pseudomolecules in the peach genome sequence (Peach v2.0; Verde et al., 2017), and the distribution of SNPs is similar to those observed for the PxF map (Figure 3), which reflect the correction of inter-pseudomolecule misassembles of the Peach v1.0 in the new version of the peach genome sequence.

A high number of skewed markers (*p* < 0.01) was mapped at the bottom part of Ad-LG5 (Figure 2). Although it has been described that the use of distorted markers could increase the genome coverage of the genetic maps (Zhang et al., 2007, 2013; Cai et al., 2015) and it may be beneficial for QTL mapping (Xu, 2008; Zhang et al., 2010), many authors eliminate the skewed markers and they are not used for linkage maps construction, diminishing the information that it is possible to obtain. It has been proposed that the study of segregation distortion is significant because distorted markers may be linked to genes or traits of interest, and these genes may be beneficial or lethal to organisms (Kirungu et al., 2019). Then, the exclusion of such markers could bias the data and result in the loss of some important genetic information. One example is the reported clustering of loci with distortion near the bottom of LG6 in sweet cherry in semi-compatible crosses, near the *Prunus* self-incompatibility *S* locus (Dirlewanger et al., 2004; Klagges et al., 2013; Guajardo et al., 2015). Until our knowledge, this is the first report of clustering of loci with high distortion in the bottom part of the LG5 in *Prunus*, and additional studies are necessary to determine the biological significance of the skewed markers in this region.

In a preliminary study in *Prunus*, two different mechanisms associated with tolerance to iron chlorosis were proposed (Gonzalo et al., 2012). The timing to overcome the lack of this essential element is the main difference between both mechanisms. The two linkage groups, LG1 and LG8, were associated with the delayed response observed in the Myrobalan plum P 2175 by Gonzalo et al. (2012). These two linkage groups were also identified in our study. However, QTLs associated with a quick response observed in LG4 and LG6 detected in “Felinem” were not identified in our study. On the other hand, the additional QTLs in LG5 and LG7 identified in “Adafuel” were not previously identified in response to iron chlorosis in this studied genotype.

A total number of 312 distinct genes were affected by significant SNPs associated with iron (Fe) deficiency-induced chlorosis, which has a large impact on yield in tree fruit crops, such as peach (Álvarez-Fernández et al., 2003; Abadía et al., 2011). However, the candidate gene PFIT, related to iron metabolism, and previously identified in Pp04 of *Prunus* (Gonzalo et al., 2012), was not identified in the candidate genes list obtained here. Peach and almond are considered to be Strategy I plants. The reduction-based strategy (Strategy I) has two main steps, the first step is the acidification of the rhizosphere that leads to an increase in the chelated Fe (III) concentration (Santi and Schmidt, 2009; Ivanov et al., 2012).

Subsequently, the root surface-localized ferric chelate reductase FERRIC-REDUCTION OXIDASE2 (FRO2; Robinson et al., 1999) reduces Fe(III) to soluble Fe(II), which is then taken up into epidermal cells by the Fe-REGULATED TRANSPORTER1 (IRT1; Eide et al., 1996). In our study, although FRO2 was not identified, two Ferric-chelate reductase genes [Prupe.1G587800 (FRO7) and Prupe.5G166400 (FRO08)] were affected by significant SNPs. Interestedly, both genes are located in different peach pseudomolecules. According to the literature, it is possible that once Fe^3+^-citrate complexes pass to the chloroplastic intermembrane space, FRO7 may reduce ferric (Fe^3+^) to ferrous iron (Fe^2+^) *via* its reductase activity (Jeong et al., 2008; Solti et al., 2016). A possible role of FRO7 in the reduction of Fe (III) to soluble Fe (II) in root Fe uptake responses to Fe-deficiency in peach should be investigated to clearly confirm this hypothesis.

Several studies using commercially available Affymetrix ATH1 GeneChips (Dinneny et al., 2008; Yang et al., 2010; Mai et al., 2016) and RNA-seq data set (Lan et al., 2013) have been carried out to study Fe deficiency and the involvement of ethylene in Strategy I plants. As a result, a total of 71 differentially expressed genes (61 being up-regulated and 10 down-regulated) overlapped between these studies. In the list of up-regulated genes was AT3G12900, which encodes a protein belonging to the 2OG-Fe(II) oxygenase family and is strongly induced by Fe deficiency. Proteins in this family are generally considered to possess oxidoreductase activity catalyzing the 2-oxoglutarate- and Fe(II)-dependent oxidation of an organic substrate using a dioxygen molecule. A previous study showed that the ethylene synthesis protein ACC oxidase also belongs to 2OG-Fe(II) oxygenase superfamily (Aravind and Koonin, 2001). In our study, the gene Prupe.1G468200 is located in Pp01 of peach and it was affected by eight significant SNPs mapped in this pseudomolecule and showing different effects, low, moderate, and modifier. This gene is an ortholog of AT1G52820 and encodes a 2-oxoglutarate (2OG) and Fe(II)-dependent oxygenase superfamily protein. The possible expression of this gene, by Fe deficiency, should be strongly verified in *Prunus* (peach or almond).

Another candidate gene was Prupe.1G541100 (ppa003848m.g), predicted as glutamyl-tRNA reductase 1, which had a modifier (3'-UTR variant) effect by a significant SNP located in Pp01 (44,202,463 bp) and mapped at 63.65 cM. The study of the transcriptional response of Arabidopsis to Fe deficiency has indicated that glutamyl-tRNA reductase (HEMA1), NYC1, and CGLD27 may represent key players in preventing photooxidative damage in Fe-deficient leaf cells (Rodríguez-Celma et al., 2013). The fast and pronounced down-regulation of HEMA1 expression suggests a rapid and dramatic decline of tetrapyrrole synthesis at an early stage of Fe deficiency. The possible implication of Prupe.1G541100 preventing photooxidative damage in Fe-deficient leaf cells in peach must be clearly confirmed.

Other important genes detected in our study were Prupe.5G039500 and Prupe.1G577000 (ppa011050.m), encoding a ferredoxin protein (FdC2) and a NIFU-like protein 2 (NIFU2), respectively. The interaction of these both genes in peach and their association with iron chlorosis response should be corroborated in further analysis. In this sense, in *Arabdopsis*, the study of the physiological and molecular changes during Fe deficiency confirmed that at the protein level, ferredoxin, the cytochrome-b6f complex, and Fe-containing enzymes of the plastid sulfur assimilation pathway were major targets of Fe deficiency, whereas other Fe-dependent functions were relatively less affected (Hantzis et al., 2018).

## Conclusion

In the present study, we have constructed, to the best of our knowledge, the first *Prunus* rootstocks linkage maps using SNPs obtained from double-digest SBG. These linkage maps were the basis to identify QTLs and putative genes related to iron chlorosis in a segregating peach–almond progeny. SNPs identified in this study present a valuable set of new SNPs identified in *Prunus* that would be useful for genetic studies in the future. Candidate genes for iron chlorosis QTL were proposed; however, future fine mapping and transcriptomic analysis are needed to enable the identification of the underlying gene(s).

## Data Availability Statement

The dataset generated for this study can be found in the NCBI-SRA database, BioProject number PRJNA773812.

## Author Contributions

VG, PM-G, and MM conceived and designed the experiments. VG, PM-G, and SS performed the experiments. VG, SS, CS, PM-G, and AC-S analyzed the data. CS and MM contributed with reagents, materials, and analysis tools. All authors contributed to the manuscript preparation, read, and approved the final version.

## Funding

This research was funded by the Spanish Ministry of Science, Innovation and Universities (MICINN) grants RFP2015-00019 and RTI2018-094176-R-C33, co-funded by ERDF A way of making EUROPE and the Regional Government of Aragon (A44 and T07-17R). It was also funded by CONICYT-REGIONAL/GORE O'HIGGINS/CEAF/R19A10003, FONDECYT 3160316, and CONICYT R16F20006 from Chile.

## Conflict of Interest

The authors declare that the research was conducted in the absence of any commercial or financial relationships that could be construed as a potential conflict of interest.

## Publisher's Note

All claims expressed in this article are solely those of the authors and do not necessarily represent those of their affiliated organizations, or those of the publisher, the editors and the reviewers. Any product that may be evaluated in this article, or claim that may be made by its manufacturer, is not guaranteed or endorsed by the publisher.
